# Mitochondrial Complex V α Subunit Is Critical for *Candida albicans* Pathogenicity through Modulating Multiple Virulence Properties

**DOI:** 10.3389/fmicb.2017.00285

**Published:** 2017-02-23

**Authors:** Shui-Xiu Li, Yan-Jun Song, Yi-Shan Zhang, Hao-Tian Wu, Hui Guo, Kun-Ju Zhu, Dong-Mei Li, Hong Zhang

**Affiliations:** ^1^The First Affiliated Hospital of Jinan UniversityGuangzhou, China; ^2^Institute of Mycology, Jinan UniversityGuangzhou, China; ^3^Department of Microbiology and Immunology, Georgetown University Medical CenterWashington, DC, USA

**Keywords:** *ATP1*, α subunit, *Candida albicans*, pathogenicity, virulence properties

## Abstract

The α subunit (*ATP1*) is a vital component of mitochondrial complex V which counts for the majority of cellular ATP production in a living organism. Nevertheless, how the α subunit influences other cellular processes such as pathogenicity in *Candida albicans* remains poorly understood. To address this question, *ATP1* mutant (*atp1*Δ/Δ) and the gene-reconstituted strain (*atp1*Δ*/ATP1*) have been constructed in this study and their pathogenicity-related traits are compared to those of wild type (WT). In a murine model of disseminated candidiasis, *atp1*Δ/Δ infected mice have a significantly higher survival rate and experience a lower fungal burden in tissues. In *in vitro* studies *atp1*Δ/Δ lose a capability to damage or destroy macrophages and endothelial cells. Furthermore, *atp1*Δ/Δ is not able to grow under either glucose-denial conditions or high H_2_O_2_ conditions, both of which are associated with the potency of the macrophages to kill *C. albicans*. Defects in filamentation and biofilm formation may impair the ability of *atp1*Δ/Δ to penetrate host cells and establish robust colonies in the host tissues. In concert with these pathogenic features, intracellular ATP levels of *atp1*Δ/Δ can drop to 1/3 of WT level. These results indicate that the α subunit of Complex V play important roles in *C. albicans* pathogenicity.

## Introduction

*Candida albicans* is the most common fungal pathogen capable of producing severe systemic infection in immune compromised individuals (Calderone et al., 2014). The overall morbidity and mortality for systemic candidiasis has been reported to be greater than 30% (Leroy et al., 2009; Wisplinghoff et al., 2014).

During hematogenous dissemination, blood-borne *C. albicans* must adhere to, colonize and invade the endothelial cells of blood vessels or the internal organs. Therefore, these cells need to develop a strategy that can counteract the stresses induced by immune-derived nutritional starvation, reactive oxygen intermediates and antimicrobial peptides (Grubb et al., 2008; Frohner et al., 2009; Walker et al., 2009). Mitochondria play an important role in each of these activities. For example, mitochondrial activity regulates the Ras1-cAMP-PKA signaling pathway that in turn induces a filamentation response for better invasion of the tissue (Dalle et al., 2010; Zhu and Filler, 2010; Grahl et al., 2015). In accordance with these morphological changes, the metabolic adaptability of fungal cells effectively facilitates the assimilation of nutrients available from a variety of sources (Brock, 2009; Fleck et al., 2011).

Mitochondrial complex V (CV) is the key enzyme in the final steps of oxidative phosphorylation. In living organisms, this ATP synthesis accounts for the majority of the cellular ATP yield required to drive the many energy-consuming reactions and processes of the organism (Senior, 1990; Harold and Maloney, 1996). This protein complex is an assemblage of complex F_1_ and complex F_0_. The former is composed of the subunits α_3_β_3_γδ𝜀 and the latter is composed of the subunits ab_2_c_10-15_. The fundamentals of oxidative phosphorylation rely on the α_3_β_3_ hexamer, of which the α subunit is encoded by *ATP1* in *C. albicans*.

Genetic abnormality of the α subunit in humans can cause a fatal infantile mitochondrial encephalopathy (Jonckheere et al., 2013). The vital roles of the α subunit are also reflected in other organisms. In *Saccharomyces cerevisiae*, deletion of the α subunit renders the organism unable to grow (Lai-Zhang et al., 1999). Knockdown of the α subunit in *Trypanosoma brucei* during its infectious stage also results in a decreased growth rate due to suppressed ATP synthesis. In *C. albicans*, the α subunit has been found at the surface of hyphae but not in yeast cells (Hernández et al., 2004). Since, the yeast-hyphae transition in *C. albicans* is known to play a role in host tissue invasion, the emergence of *ATP1* on the hyphal form gives rise to the question of what the α subunit contributes to *C. albicans* pathogenicity *in vivo* and how the essential energetic pathway gets involved with fungal growth in the host.

To answer these questions, we construct a null *ATP1* mutant (*atp1*Δ/Δ) and a gene-reconstituted strain (*atp1*Δ*/ATP1*). The α subunit (*ATP1*) of *C. albicans* was characterized from these strains and compared to the wild strain (WT), and pathogenic roles of *ATP1* were then firstly analyzed *in vivo*. We demonstrate that deletion of *ATP1* diminishes the ability of *C. albicans* to cause systemic infections in animal model. The attenuated pathogenicity in these mutants may be due to some defects in the process of host invasion and compromised capability to form biofilm. We conclude that the α subunit is essential for *C. albicans* pathogenicity.

## Materials and Methods

### Strains and Growth Conditions

A null mutant of *ATP5* (*atp1*Δ/Δ) was constructed by PCR-mediated homologous recombination as described previously (Sasse and Morschhäuser, 2012). *C. albicans* SC5314 (wild type; Gillum et al., 1984) was used to generate the *atp1*Δ/Δ mutant strain (*atp1*Δ*::FRT*/*atp1*Δ*::FRT*) and *atp1*Δ*/ATP1* gene-reconstituted strain (*atp1*Δ*::FRT* /*ATP1*::FRT). The primers used for gene deletion in this study are listed in Supplementary Table S1. The confirmation of the *atp1*Δ/Δ mutant and reconstituted strain (*atp1*Δ*/ATP1)* are performed by PCR amplicons using primer pairs as shown in Supplementary Figures S1 and S2. Strains were routinely grown in YPD broth or on YPD agar (1% yeast extract, 2% peptone, and 2% glucose) with or without compounds as indicated.

### Effect of *ATP1* on the Virulence in Mice

A mouse model of disseminated candidiasis was used to evaluate the virulence of the strains (Spellberg et al., 2003, 2005). Female BALB/c mice (18–22 g; Guangdong Medical Laboratory Animal Center, Foshan, Guangdong, China) were used for all experiments. Mice were injected via the lateral tail vein with either a suspension of 1 × 10^5^ cells or 1 × 10^6^ cells from each strain. Survival rate was calculated from 10 infected mice per strain. For determination of fungal burden, another three mice from each group were euthanized after 1, 24, 48, and 72 h infection. Kidney, spleen and liver were harvested, weighed, homogenized, and quantitatively cultured. In addition, at day 1 of infection, mice were killed and organs removed to fix in 10% buffered formalin, then embed in paraffin, sectioned and stained with **P**eriodic **A**cid-**S**chiff for histological study. Mortality was represented with Kaplan–Meier survival curves and quantitative tissue burdens were marked in the log scale and compared in the Mann–Whitney test.

### Ethics Statement

The animal experiments were performed under the guidance of a protocol approved by the Animal Study Committee of the Institute of Dermatology, CAMS, according to the National Guidelines for Animal Care. All animal experiments were carried out with permission from the Ethical Committee of Institute of Zoonosis, Jinan University, Guangdong, China (Ref no. 20080101).

### Interaction between *C. albicans* and Macrophages

The mouse macrophage cell line RAW264.7 (ATCC) was used for all assays. To investigate the susceptibility of *C. albicans* strains to the macrophages, macrophage cells were seeded in a 96-well plate at 5 × 10^4^ cells/ml in 150 μl of DMEM with 10% FBS overnight at 37°C in 5% CO_2_. The *C. albicans* were diluted into DMEM at 2 × 10^6^ cells/ml, and 50 μl was added into the first column wells, mixed and then serially diluted 1:4 a total of six times. Plates were incubated at 37°C and 5% CO_2_ for 24 h. Colonies were counted. The survival rate was calculated as the numbers of colonies with macrophages divided by the number of colonies in the absence of macrophages. *P*-values were determined using the unpaired Student’s *t*-test (She et al., 2015). For evaluating macrophage survival, macrophage cells were seeded in 24-well plate at 1 × 10^6^ cells in 1 ml of DMEM with 10% FBS, and cultured at 37°C and 5% CO_2_ for 24 h. The *C. albicans* cells were suspended in DMEM with 10% FBS to 1 × 10^6^ cells/ml, then added into the wells. After 1 h of co-incubation, supernatant was removed from each wells, washed and stained with Hoecheset33258 for 1 h, and observed by a fluorescent microscope (Olympus, Japan) with a TRIT-C/Texas red filter set. Hoecheset-positive (damaged) and total macrophages were counted, and the percentage of damaged macrophages was calculated as the number of damaged macrophages divided by that of total macrophages (Yu et al., 2014).

### Effect of *ATP1* on Endothelial Cells Damage

The extent of damage of endothelial cells by the *C. albicans* was quantified by measuring lactate dehydrogenase (LDH) activity (Mayer et al., 2012). The human umbilical vein endothelial cell line HUVEC (ATCC CRL-1730, LGC Standards, Promocell) cells were seeded in a 96-well plate at 1 × 10^5^ cells/ml in 200 μl of DMEM with 10% FBS overnight at 37°C and 5% CO_2_ for 2 days. Cells were washed and 100 μl DMEM with 2% FBS were added. The *C. albicans* were diluted into DMEM at 5 × 10^5^ cells/ml, and 100 μl was added to infect endothelial cells. Incubation was carried out at 37°C and 5% CO_2_ for 15 or 24 h. Measurement of LDH activity with the Cytotoxicity Detection Kit was performed according to the manufacturer’s manual. Absorbance of the samples was measured at 490 nm. Medium only and low control values were subtracted from all sample values. Damage was expressed as percentage of the control strain. Each experiment was performed at least in triplicate.

### Effect of *ATP1* on *C. albicans* Adhesion

The adhesion was observed visually (Nobile et al., 2006). The 24-well flat bottomed pre-sterilized microtiter plates were incubated in fetal bovine serum overnight at 37°C. A suspension of 1 × 10^7^ cells of *C. albicans* in Spider media was added to the wells and incubated at 37°C for 2 h. Non-adherent cells were removed by washing with PBS. Fresh Spider media was added to the corresponding wells and the plates were incubated at 37°C for 24 h. The wells were washed and photographed.

### Effect of *ATP1* on *C. albicans* Filamentation

A 1 × 10^6^ cells/ml suspension of each strain in YPD, YPD+10% FBS, Spider and Lee’s liquid media was added to the wells of a 12-well flat-bottomed pre-sterilized microtiter plate and incubated at 37°C for 2 h. The plates were visualized under inverted microscope and photographed. In addition, cells of each strain were serial diluted and spotted on YPD, YPD+10% FBS, Spider, Lee’s and SLAD agar, and incubated at 37°C for 7 days. The colonies were photographed.

### Effect of *ATP1* on *C. albicans* Biofilm Formation

The biofilms were observed by Confocal laser scanning microscopy (CSLM) (Nobile et al., 2006). Briefly, all strains were grown in Glass Bottom Cell Culture Dish. After 24 h of incubation at 37°C, the resulting biofilms were washed and stained with 25 μg/ml Concanavalin A-Alexa Fluor 594 conjugate (C-11253; Molecular Probes, Eugene, OR) at 37°C for 1 h. CSLM was performed with a Zeiss LSM 510 upright confocal microscope (Carl Zeiss, Thornwood, NY, USA), using a Zeiss Achroplan 40×, 0.8-W objective, and a HeNe1 laser with an excitation wavelength of 543 nm. Moreover, biofilms activity was assessed by XTT reduction assay and the crystal violet assay, accordingly to previously described protocols (Peeters et al., 2008; Pierce et al., 2008; Krom and Willems, 2016). Each experiment was performed at least in triplicate.

### Quantitative Real Time Polymerase Chain Reaction (qRT-PCR) Assay

Overnight cultures of tested strains in 5 ml of YPD at 30°C were collected, then diluted into 100 ml of YPD or Spider medium to obtain an OD_600_ of 0.2 and incubated at 37°C for additional 6 h. Total RNA was extracted using the E.Z.N.A. Yeast RNA kit (Omega Bio-tek) following the manufacturer’s instruction. cDNA was synthesized and qRT-PCR was done as previously described (Guo et al., 2014).

### Stress Susceptibility

To investigate the sensitivity of *C. albicans* strains to different stress agents, cells were overnight cultured, washed and suspended in PBS with an initial OD_600_ of 1.0. Five microliters of 10-fold dilutions was spotted onto YPD agar without or with different stress agents. For testing the sensitivity of *C. albicans* strains to heat stress, plates were cultured at indicated temperatures for 2 days and then photographed. The minimum inhibitory concentrations (MICs) were determined for each strain according to the broth microdilution method of the Clinical and Laboratory Standards Institute (CLSI) M27-A3 protocol (Method M27-A3 CLSI, 2008).

### Growth Curve Assay

All strains were routinely grown in 5 ml of YPD at 30°C overnight, washed with PBS, then inoculated in 100 ml of YPD with an OD_600_ of 0.02. Shake cultures were grown at 30°C and OD_600_ of each strain was measured every two hours.

### Mitochondrial Function Assay

All strains were grown in YPD overnight at 30°C. Mitochondrial complex V activity was measured by an assay kit according to the manufacturer’s protocol. For determinations of intracellular ATP concentrations, an aliquot of 1 × 10^6^ cells from each strain was mixed with the same volume of BacTiter-GloTM reagent (Promega Corporation, Madison, WI, USA) and incubated for 5 min at room temperature as described previously (Zhang et al., 2013). ROS measurement was performed by using a oxidation-sensitive fluorescent dye DCFDA (Guo et al., 2014). Briefly, a suspension of 2.5 × 10^6^ cells was stained with DCFDA (20 μg/ml) at 37°C for 20 min. The emission spectra at 488 nm and excitation spectra at 595 nm were determined by FACScan flow cytometer (Becton Dickinson). The cyanine dye JC-1 was used for determination of mitochondrial membrane potential of each strain (She et al., 2015). A suspension of 2.0 × 10^6^ cells was incubated with 5 μM JC-1 at 37°C for 15 min and excitation spectra at 595 nm was determined by FACScan flow cytometer with emission spectra at 488 nm (Becton Dickinson).

## Results

### Deletion of *ATP1* Results in Avirulence in a Mouse Model of Hematogenously Disseminated Candidiasis

**FIGURE 1:**
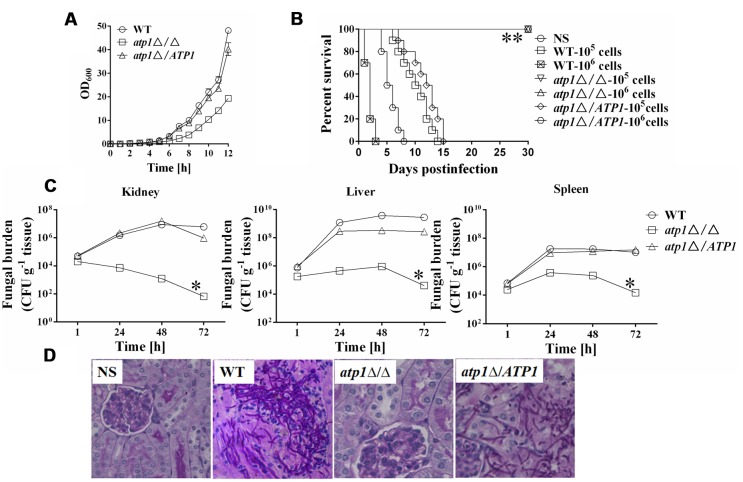
**Deletion of *ATP1* results in avirulence in a mouse model of hematogenously disseminated candidiasis. (A)** Reduced growth rate of the *atp1*Δ/Δ was determined by measuring the OD_600_ of cells growing in YPD at 30°C. **(B)** Mice survival following infection with 1 × 10^5^ and 1 × 10^6^ cells of the *Candida albicans*. ^∗∗^ *P* < 0.0001 compared to mice infected with WT. **(C)** Fungal burden (CFU/g tissue) of mice infected with 1 × 10^6^ cells of the *C. albicans*. Following infection, at 1, 24, 48, and 72 h, kidney, liver and spleens were removed, weighed, homogenized and samples plated on YPD agar medium. The total CFUs in tissue for each strain were shown over time. Data are from two experiments with three mice per time point for each strain (mean ± SD). ^∗^*P* < 0.05 compared to mice infected with WT. **(D)** Periodic acid Schiff staining of kidney sections from mice after 1 day of infection with *C. albicans*. Pictures were taken at 40× magnification.

The *atp1*Δ/Δ mutant displayed moderately reduced growth rate *in vitro* even when grown in rich medium (**Figure 1A**, Supplementary Table S3), which is similar to other *C. albicans* mutants with reduced virulence (Murad et al., 2001; Hwang et al., 2003; Chauhan et al., 2005; Lopes da Rosa et al., 2010; Chang et al., 2015; Lee et al., 2015). We speculate that such slow growth of *atp1*Δ/Δ *in vitro* may affect its virulence. A murine model of hematogenously disseminated candidiasis was first used to measure virulence changes in *atp1*Δ/Δ. All of the mice infected intravenously with either 1 × 10^5^ cells or 1 × 10^6^ cells of *atp1*Δ/Δ survived post 30 days infection (**Figure 1B**). By contrast, mice infected with WT began to die on days 7 and 1 post infection, and all mice were moribund on days 14 and 3, respectively. On the other hand, mice infected with 1 × 10^5^ cells and 1 × 10^6^ cells of *atp1*Δ*/ATP1* began to die on days 8 and 4 and all mice were moribund on days 15 and 8, respectively. Clearly, *ATP1* is required for *C. albicans* virulence, and the loss of virulence in *atp1*Δ/Δ is correlated with is not slow growth *in vitro*.

Next, to correlate the avirulence of *atp1*Δ/Δ with its growth in host tissues, the fungal load was evaluated in kidney, liver and spleen (**Figure 1C**). The colony forming unit (CFU) was the same for all strains 1 h post infection. However, tissue loads in either WT or *atp1*Δ*/ATP1* increased significantly (by about 2 orders of magnitude) in kidney while *atp1*Δ/Δ load gradually reduced post infection and dropped by about one order of magnitude at 48 h post infection. The fungal loads in liver and spleen reached their peaks at 48 h for all three strains but reduced fungal cell populations were observed in mutant during 72 h post infection when compared to the control strains. At 72 h post infection with *atp1*Δ/Δ, fungal cells continued to be cleared from the kidney, liver and spleen. The significant reduction of fungal burden in the kidneys infected with the *atp1*Δ/Δ within 3 days of infection suggests that *ATP1* is required for kidney invasion and is necessary for the organism to persist in other tissues at later time points.

The low fungal burden in the kidneys of mice infected with the *atp1*Δ/Δ was verified by histopathology. After 24 h of infection, the kidneys infected with *atp1*Δ/Δ were scant to detect in tissue sections, as demonstrated by a absence of fungal hyphae and inflammatory cells, even though the fungal load was still positive according to CFU count. By contrast, kidneys infected with either WT or *atp1*Δ*/ATP1* displayed the expected micro-abscesses consisting of numerous inflammatory cells surrounded by abundant hyphae or pseudohyphae (**Figure 1D**), suggesting that the avirulence of the *atp1*Δ/Δ is likely due to defects in tissue invasion and colonization ability.

### Deletion of *ATP1* Results in Hypersensitivity to Macrophages

**FIGURE 2:**
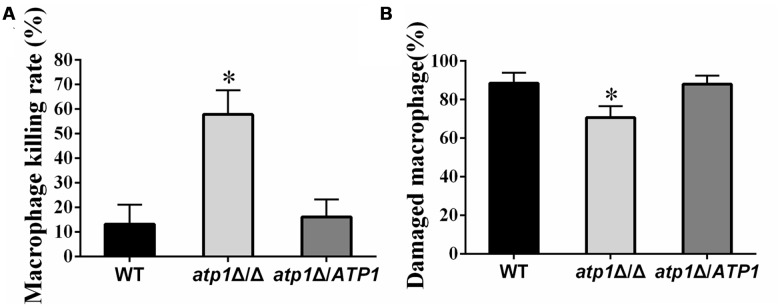
**Interaction between *C. albicans* and macrophages. (A)** Viability of all strains is shown after an overnight incubation with macrophages (RAW264.7). Viable colonies of each strain were counted under microscopy. Data are averages of three separate experiments. ^∗^*P* < 0.05 compared with WT. **(B)** Macrophages were incubated with the indicated strains of *C. albicans* for 1 h and the extent of macrophages damage was then determined. Data are averages of three separate experiments. ^∗^*P* < 0.05 compared with WT.

Macrophages play a key role in host defense against *C. albicans* infections (Mayer et al., 2012). In order to invade the host organs and begin colonization, blood-borne *C. albicans* must evade the attacks from the immune system prior to crossing the endothelial cell barrier of blood vessels (Grubb et al., 2008). We therefore investigate whether *ATP1* is required for *C. albicans* sensitivity to host killing events. The sensitivity of the *atp1*Δ/Δ to macrophages and its capability to damage macrophages were both measured. Macrophage killing tests revealed that *atp1*Δ/Δ is more vulnerable to macrophages, as demonstrated by the survival of fewer *atp1*Δ/Δ cells after an overnight incubation with macrophages than those from either WT or *atp1*Δ*/ATP1* (**Figure 2A**). On the other hand, far fewer macrophages were damaged when incubated with *atp1*Δ/Δ cells than when incubated with the control strains (**Figure 2B**). Evanescence of *atp1*Δ/Δ cells and insufficient killing of macrophages suggest that the *ATP1* is required for *C. albicans* to evade macrophage attacks.

### Deletion of *ATP1* Disables Utilization of Non-glucose Carbon Sources and Increases Sensitivity to Oxidative Stress in *C. albicans*

**FIGURE 3:**
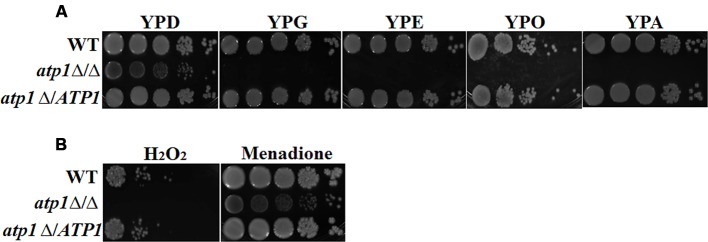
**Deletion of *ATP1* disables utilization of non-glucose carbon sources and elevates oxidative stress. (A)** *C. albicans* strains were grown overnight in YPD medium, washed and serially diluted in PBS. Three microlitres of each serial dilution was plated on the YP medium supplemented with 2% glucose (YPD), 2% glycerol (YPG), 3% oleic acid (YPO), 2% ethanol (YPE), or 2% acetate (YPA) agar plates. **(B)** Oxidative stress response. Strains were grown in the presence of 8 mM hydrogen peroxide and 0.05 mM menadione. All cultures were incubated for 2 days at 30°C.

The capability of *C. albicans* to utilize non-glucose carbon sources and thereby counteract elevated reactive oxygen species (ROS) within macrophages are believed to be two elemental factors for the establishment of candidiasis (Frohner et al., 2009). We find that growth of the *atp1*Δ/Δ on spot assay is slightly less pronounced than those of WT and *atp1*Δ*/ATP1* on YPD agar, but show no growth on YPG (glycerol), YPE (ethanol), YPO (oleic acid), or YPA (acetate). The last four media are YP-based media integrated with a non-glucose carbon source (**Figure 3A**). For oxidative stress response, the *atp1*Δ/Δ showed a reduced growth when exposed to H_2_O_2_, but no response to menadione (**Figure 3B**). These data indicate a vulnerable response of the *atp1*Δ/Δ to macrophage attacks may be due largely to an inability to consume non-glucose carbons as well as poor management of oxidative stress.

### Deletion of *ATP1* Results in Less Damage to Endothelial Cells *in vitro*

**FIGURE 4:**
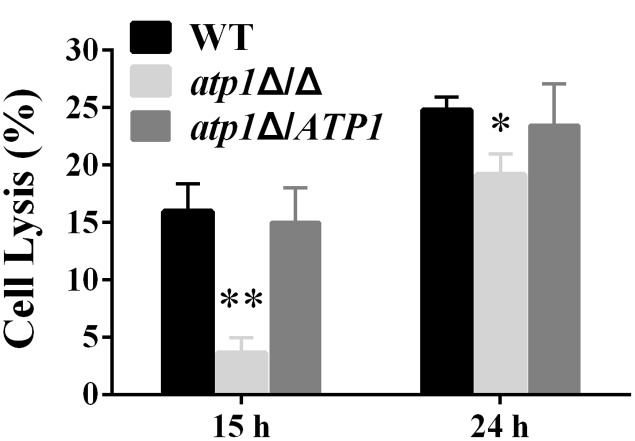
**Deletion of *ATP1* causes less damage of human endothelial cells.** Monolayers of HUVEC endothelial cells were infected with *C. albicans* strains for 15 or 24 h. Damage of host cells was determined by measuring lactate dehydrogenase (LDH) levels. Results are the mean ± SD of at least three independent experiments, each performed in triplicate. ^∗^*P* < 0.05 and ^∗∗^*P* < 0.01 compared to WT.

Endothelial cells of blood vessels provide a physical barrier for *C. albicans* to penetrate and invade the internal organs. We find that the *atp1*Δ/Δ has a significantly reduced capacity to destroy endothelial cells. The cell lysis rates were 77% less and 24% less at 15 and 24 h of infection (**Figure 4**), respectively, when compared to WT. These results indicate that *ATP1* is required for endothelial cell penetration.

### Deletion of *ATP1* Displays Defective Adhesion and Invasion

**FIGURE 5:**
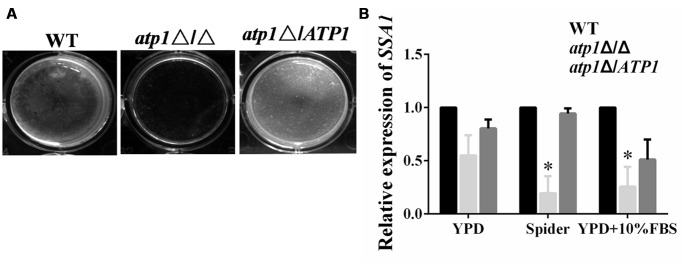
**Deletion of *ATP1* displays defective adhesion and invasion. (A)** Effect of *ATP1* on *C. albicans* adhesion. 1 × 10^7^ cells suspension of *C. albicans* in spider media was added to 24-well flat bottomed plates and incubated for 24 hours at 37°C. The wells were washed with PBS and taken photos. **(B)** Effect of *ATP1* on the expression of *C. albicans* adhesion gene. Effect of *ATP1* on the expression of *C. albicans* invasion gene. *C. albicans* cells were incubated in YPD, Spider, YPD+10%FBS media at 37°C for 6 h. Following incubation expression of the indicated genes were determined by qRT-PCR. Expression level of each gene is displayed after normalization with internal control housekeeping gene 18S. The histogram shows the relative expression fold change of genes. Results represent the average of three independent experiments ±SD. ^∗^*P* < 0.05 when compared with WT.

The contribution of *ATP1* to adhesion events has also been tested in microtiter plates *in vitro* in order to understand whether less lysis of macrophages or endothelial cells may be due to the inefficiency of adhesion (Filler et al., 1991; Park et al., 2005; Wächtler et al., 2011). Adhesion assay reveals that *atp1*Δ/Δ fails to adhere to the bottom of the microtiter plate when compared with WT (**Figure 5A**). In qRT-PCR assay, the gene expression levels of *SSA1* and *ALS3* were markedly down-regulated in the *atp1*Δ/Δ when compared with WT (**Figure 5B**). These genes have long been known for their prominent roles in host tissue adhesion and invasion (Phan et al., 2007; Sun et al., 2010).

### Deletion of *ATP1* Displays Defective Filamentation and Colony Formation

**FIGURE 6:**
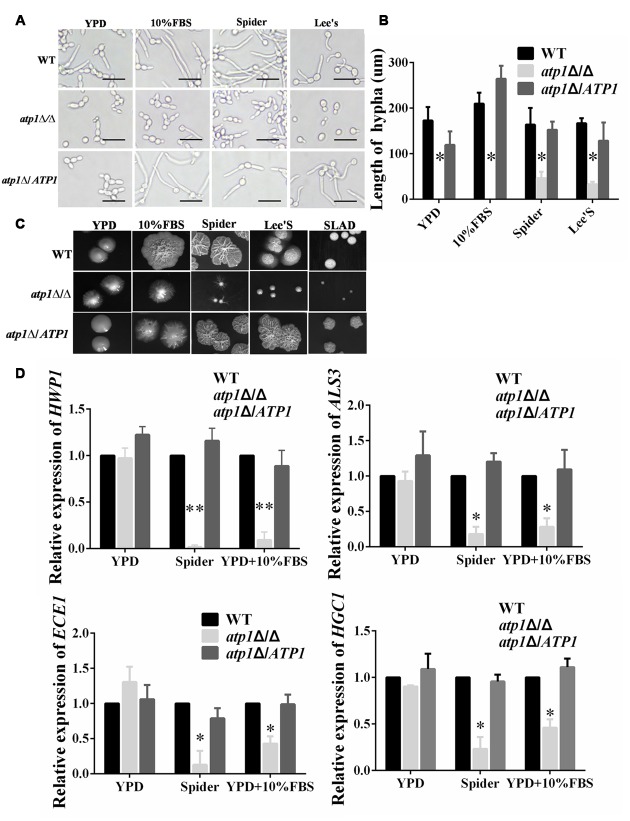
**Deletion of *ATP1* displays defective filamentation and colony formation. (A)** Overnight cultures of the strains were resuspended to 1.0 × 10^6^ cells/ml, and added into 12-well microtiter plates with filaments-inducing media. All the plates were cultured at 37°C for 2 h before photographed. **(B)** Length of filaments was measured. **(C)** Overnight cultures of the strains were resuspended, and 500 cells were spotted onto the indicated filaments-inducing agar plates. All the plates were cultured at 37°C for 7 days before photographed. **(D)** Effect of *ATP1* on the expression of *C. albicans* hyphal-specific genes. *C. albicans* cells were incubated in YPD, Spider, YPD+10%FBS media at 37°C for 6 h. Following incubation expression of the indicated genes were determined by qRT-PCR. Results represent the average of three independent experiments ± SD. ^∗^*P* < 0.05 and ^∗∗^*P* < 0.01 when compared with WT.

Filamentous growth enables *C. albicans* to penetrate host immune cells and tissue more efficiently after endocytosis (Phan et al., 2000; Dalle et al., 2010; Zhu and Filler, 2010). The requirement of *ATP1* for filamentation has been tested under hyphal inducing conditions. In each hyphal inducing medium (10% FBS, Spider and Lee’s medium), the *atp1*Δ/Δ displays shorter or even no filamentous growth at 2 h growth point compared with WT, which has massive and long filamentous growth under microscopy (**Figures 6A,B**, Supplementary Figure S3). Similar results were also present on each agar medium. The *atp1*Δ/Δ displays a more severe defective phenotype in filamentous growth and forms small downy or even smooth colonies which lack the peripheral and invasive filaments, while WT produced large colonies with florid and invasive filaments on the edges (**Figure 6C**). The *atp1*Δ*/ATP1* has intermediate effects in these matters between WT and *atp1*Δ/Δ. The filamentation defect in the *atp1*Δ/Δ is consistent with marked down-regulation of hyphal-specific genes such as *HWP1, ALS3, ECE1, HGC1* in qRT-PCR test (**Figure 6D**). These results indicate that *ATP1* is required for the filamentation process in *C. albicans*.

### Deletion of *ATP1* Displays Defective Biofilm Formation

**FIGURE 7:**
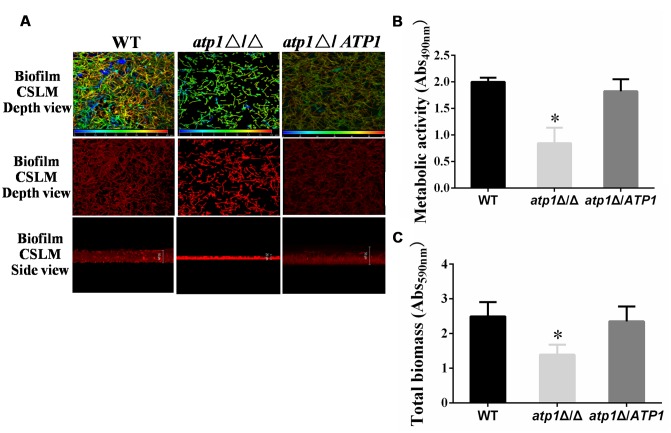
**Deletion of *ATP1* displays defective biofilm formation. (A)** Biofilms of strains were stained with Concanavalin A-Alexa Fluorconjugate and analyzed by CSLM visualization. The top two panels show the visual appearance and depth over top view, in which blue color represents cells close to the glass bottom and red color represents cells far from the glass bottom. The bottom panel is side view of biofilm depth. At depth views, WT, blue = 0 μm and red = 45 μm; *atp1*Δ/Δ, blue = 0 μm and red = 16 μm;*atp1*Δ*/ATP1*, blue = 0 μm and red = 55 μm. At the side views, the biofilm depths are 50 μm for WT and *atp1*Δ*/ATP1* but 20 μm for *atp1*Δ/Δ. **(B)** Biofilm was quantified colorimetrically by XTT assay, which measured biofilm metabolic activity. **(C)** Biofilm was quantified colorimetrically by crystal violet assay, which measured biofilm total biomass. Error bars represent the standard deviation among results for different isolates. ^∗^*P* < 0.05 when compared with WT.

Aside from filament propensity, *Candida* biofilm also carries important clinical consequences because it will encase organism from antifungal therapy and withstand host immune defense (Douglas, 2003; Nett et al., 2010). Confocal scanning laser microscopy (CSLM) imaging was used to visualize biofilm formation in the *atp1*Δ/Δ and meanwhile, the biomass and cell metabolic activity within biofilm were measured. After 24 h culture, the *atp1*Δ/Δ forms a rudimentary biofilm with a thickness of less than 20 μm, while WT produces biofilm with thickness of over 50 μm, and *atp1*Δ*/ATP1* produces biofilm of intermediate thicknesses (>20 μm and <50 μm) (**Figure 7A**). Under CSLM, the rudimentary *atp1*Δ/Δ biofilm consists mainly of yeast cells, with few filaments, whereas the biofilms of WT and *atp1*Δ*/ATP1* have abundant filaments (**Figure 7A**). Consistently, the metabolic activity and total biomass of the *atp1*Δ/Δ are obviously reduced by XTT assay (**Figure 7B**) and by crystal violet assay, respectively when compared with WT and *atp1*Δ*/ATP1* (**Figure 7C**). These results suggest that *ATP1* deletion may be responsible for the failure of *C. albicans* to form biofilms.

### Deletion of *ATP1* Results in a Suppression of Mitochondrial Activity

**FIGURE 8:**
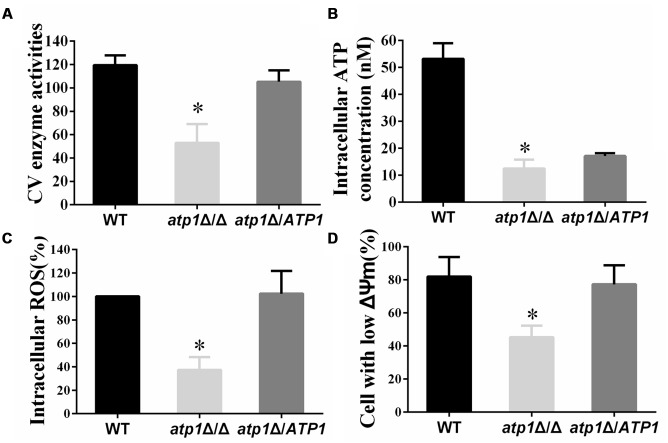
**Deletion of *ATP1* results in decreasing mitochondrial activity. (A)** Mitochondrial Complex V enzyme activities. **(B)** The intracellular ATP content was measured by microplate reader. **(C)** The ROS levels were measured with DCFDA dye by flow cytometry. Cells were collected, resuspended in PBS and incubated with DCFDA at 37°C for 20 min. **(D)** Mitochondrial membrane potential was measured with JC-1 dye by flow cytometry. Cells were collected, resuspended in PBS and incubated with JC-1 for 15 min at 37°C. Gated region R1 includes cells with intact mitochondrial membranes and gated region R2 depicts cells with loss of mitochondrial membrane potential. ^∗^*P* < 0.01.

The association of mitochondrial activity with *C. albicans* filamentation has been noted previously (Bambach et al., 2009; Grahl et al., 2015). Enzymatic assay of mitochondrial CV and intracellular ATP content were used to evaluate mitochondrial activity in the *atp1*Δ/Δ mutant. In the absence of the α subunit of Complex V, we find that the activity of Complex V is only 50% of the WT level (**Figure 8A**) and intracellular ATP drops to 1/3 of WT levels (**Figure 8B**). Meanwhile, the membrane potential of the *atp1*Δ/Δ is significantly decreased to nearly 50% of WT (**Figure 8D**). These data therefore confirm a critical role of *ATP1* on mitochondrial function. However, in spite of these suppressed mitochondrial functions, we find that *atp1*Δ/Δ has a decreased ROS when compared with the WT (**Figure 8C**). Unlike other mitochondrial CI mutants with high ROS (Li et al., 2011; She et al., 2015), such reduced ROS levels in *atp1*Δ/Δ may be due to the fact that CV is not a direct electron acceptor. However, the contribution of ROS scavengers in *atp1*Δ/Δ needs to be clarified with further study.

### Deletion of *ATP1* Changes the Stress Response in *C. albicans*

**FIGURE 9:**
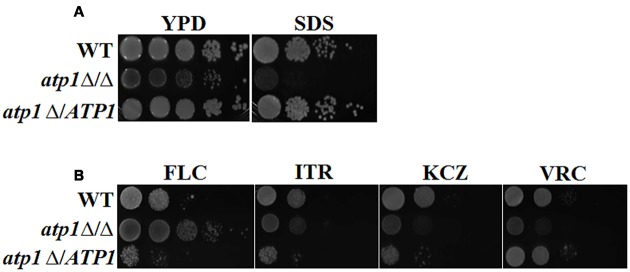
**Deletion of *ATP1* diverge stress response. (A)** *C. albicans* strains were grown overnight in YPD medium, washed and serially diluted in PBS. Three microlitres of each serial dilution were grown in the presence of 0.06% SDS. **(B)** Susceptibility to different antifungal agents. Strains were grown in the presence of 2 μg/ml FLC, 2 μg/ml ITR, 2 μg/ml KCZ, and 2 μg/ml VRC.

The fate of invasive *Candida* cells also depends on how well they handle stress in the host environment. To evaluate these adaptive skills, we measured the susceptibility of the *atp1*Δ/Δ to various stresses. As shown in **Figure 9A**, *atp1*Δ/Δ is susceptible to plasma membrane stress (SDS), but the susceptibilities to cell wall (Calcofluor White, Congo Red), heat, calcium (CaCl_2_) and osmotic stress (NaCl) are less pronounced in mutant when compared to its growth on YPD agar (Supplementary Figure S4). Interestingly, *atp1*Δ/Δ shows resistance to FLC, but not to ITR, KCZ and VRC (**Figure 9B**, Supplementary Table S2) even though the membrane of this mutant is more susceptible to SDS. The FLC resistance in the absence of *ATP1* highlights the possible link between *ATP1* function and FLC target or uptake process.

### The Expression of *ATP1* in Yeast and Hyphal forms of *C. albicans*

In *C. albicans*, the α subunit has been found at the surface of hyphae but not in yeast cells (Hernández et al., 2004). To better understand the pathogenic roles of *ATP1* in this organism, we also measured the gene expression levels of *ATP1* at the yeast and hyphal stages of WT. We find *ATP1* expression levels at the hyphal form are similar to those of yeast form (Supplementary Figure S5). Likely, one possible explanation is that Atplp is regulated at the post-translational level rather than transcriptional level. Whether *ATP1* is hyphal-specific requires further investigation.

## Discussion

The previous studies have demonstrated that mitochondrial activity is prerequisite for *C. albicans* virulence (Bambach et al., 2009; Grahl et al., 2015). In this study, the α subunit of mitochondrial Complex V was deleted from *C. albicans* and its pathogenic association was firstly assessed in animal model and *in vitro* immune cell lines. Our results with the *atp1*Δ/Δ confirm that *ATP1* is required for *C. albicans* virulence by interfering with adhesion, filamentation, biofilm formation and stress adaptation.

*Candida albicans* mutants with slow growth *in vitro* often show defects in virulence as well, but this is not always true (Noble et al., 2010). Clearly, the *atp1*Δ/Δ grows more slowly *in vitro.* In order to minimize this slower growth effect, mice infected with ten times more *atp1*Δ/Δ cells were also included for virulence study. No matter how many fungal cells were introduced, all mice infected with the mutant survived without any clinical signs of the disease, whereas mice infected with WT were always killed. It is therefore likely that the loss of virulence in the *atp1*Δ/Δ is not simply due to slow growth, but rather that the lack of *Candida* cells in the kidneys of the *atp1*Δ/Δ infected mice is due to the defects of the mutants in colonizing and invading the host tissues.

After reaching the bloodstream, *C. albicans* must evade immune attacks and then penetrate the endothelial cells of the blood vessels (Mayer et al., 2012). The results of interactions between *C. albicans* and macrophages show that the *atp1*Δ/Δ is more vulnerable to be killed by macrophages. Among the macrophages, highly reactive intermediates like ROS that are generated during interaction with pathogens, and nutrient starvation are believed to be the strategies used by macrophages to kill *C. albicans* (Frohner et al., 2009). Obviously, the *atp1*Δ/Δ has no advantages against macrophages, which are demonstrated by no growth of the *atp1*Δ/Δ on non-glucose carbon source agar as well as increased sensitivity to H_2_O_2_. These findings may also explain an avirulent phenotype of the mutant. On the other hand, stress response is almost intact with the *atp1*Δ/Δ mutant, except that it is susceptible to SDS and resistant to FLC. Unlike Complex I subunit mutants that often display a hypersensitivity to FLC (Sun et al., 2013), the resistance to FLC over other azoles suggests that *ATP1* may be specific for FLC target binding or uptake processes.

Once *C. albicans* cells survive the attacks of the immune cells, the next step is for them to penetrate the endothelial lining of the blood vessels and then colonize the internal organs. The defects of the *atp1*Δ/Δ in adhesion, invasion, and damage to endothelial cells may reflect an impaired capacity to disseminate through blood vessels during systemic candidiasis. Indeed, *C. albicans* mutants which have defects in invading and damaging endothelial cells frequently display attenuated virulence during candidiasis in mice (Phan et al., 2000; Sanchez et al., 2004; Chiang et al., 2007; Nobile et al., 2008). Another factor to affect tissue invasion is hyphal formation (Dalle et al., 2010; Zhu and Filler, 2010), which is also required for *C. albicans* escaping from host macrophages after phagocytosis (Lorenz et al., 2004; McKenzie et al., 2010). Filamentation is the major virulence factor of candidiasis (Lo et al., 1997; Mitchell, 1998). One of the more striking phenotypes of the *atp1*Δ/Δ is its short filaments under microscopy and minimal colonies on hyphae-inducing media. To date, the direct link between mitochondrial function and morphological switches in *C. albicans* is still unknown; however, some have suggested that mitochondrial activity regulates the Ras1-cAMP-PKA signaling pathway that is responsible for the yeast-hyphae transition (Grahl et al., 2015).

Biofilm formation provides a useful platform for *C. albicans* to form robust colonies in the host tissues and protect *C. albicans* from environmental stresses, such as antifungal agents, oxidative stress and immune attacks (Douglas, 2003; Seneviratne et al., 2008). Moreover, the filaments in biofilm have a strong propensity for tissue invasion (Nett et al., 2010). By consequence of defective filamentation, CSLM imaging reveals that the *atp1*Δ/Δ forms a rudimentary biofilm that is composed of yeast cells in contrast to a mature biofilm of WT that is made of yeast, pseudohyphae and hyphae. Therefore, the defects of the *atp1*Δ/Δ on hyphal formation and biofilm may further degrade the virulence of *C. albicans* in a mice disseminated model.

Atp1p has been found at the surface of hyphae but not in yeast cells (Hernández et al., 2004). Its antigenic property has also been convincingly shown by the detection of Atp1 antibodies both in human sera from systemic candidiasis patients and in serum from mice systemically infected with *C. albicans* (Pitarch et al., 2004; Martínez-López et al., 2008). Our observation on defective hyphae of the *atp1*Δ/Δ aligns well with the enrichment of Atp1p at the hyphal phase. However, *ATP1* expression in the hyphal form of WT is the same as for the yeast form in this study. Also, the experiments regarding mitochondrial function in this study were all carried out in *Candida* cells grown in YPD (yeast phase growth), and we speculate that the dysfunctional mitochondria of the mutants would not exclude the possible roles of *ATP1* in the yeast growth phase as well. In fact, such a possibility is currently being investigated.

The critical roles of *ATP1* in cellular activity have been described previously. In a large scale gene annotation study with transposon mutants, haploinsufficiency of this gene resulted in detectable phenotypes (Oh et al., 2010). This agrees with our findings that intracellular ATP level in reconstituted strain is as low as gene deletion mutant. However, most phenotypes of *atp1*Δ*/ATP1* observed in this study are either compatible to WT or in a manner of intermediate effect, the mechanism of such haploinsufficiency is not yet clear.

In summary, we first identified that the α subunit is essential for pathogenicity in *C. albicans.* The avirulence phenotype of the *atp1*Δ/Δ may be caused by a combination of decreased sensitivity to macrophages, together with a reduced capacity to damage endothelial cells and defects in filamentation and biofilm formation.

## Author Contributions

SL, YS, and YZ contributed equally to the article; SL, YS, and HZ designed the research plan; SL, YS, YZ, and HW executed the experiments. SL, YS, YZ, HW, HG, KZ, DL, and HZ performed the data analyses and writing. All authors read and approved the final manuscript.

## Conflict of Interest Statement

The authors declare that the research was conducted in the absence of any commercial or financial relationships that could be construed as a potential conflict of interest.
