# Supplementary material for: Mitochondrial Complex V α Subunit Is Critical for Candida albicans Pathogenicity through Modulating Multiple Virulence Properties
Source: Front Microbiol. 2017 Feb 23;8:285. doi: 10.3389/fmicb.2017.00285 (PMC5322696; doi:10.3389/fmicb.2017.00285)
Supplement: Supplementary file 1 [file Data_Sheet_1.DOCX]

**Table S1｜**Primers used in this study for the deletion of *ATP1* in *C. albicans.*

| Primers |  | Sequence ^a^ |  | Reference |
| --- | --- | --- | --- | --- |
| ATP1-1 |  | 5’-CCCgggcccTCAGTCTATCAAACCGAAGT -3’ |  | This study |
| ATP1-2 |  | 5’-CCCctcgagTCTGGCAGCAATAGTAGC -3’ |  | This study |
| ATP1-3 |  | 5’-ATAAGAATgcggccgcTATTAAGACCAAGGGTGA-3’ |  | This study |
| ATP1-4 |  | 5’-TCCccgcggCTTACTTACGAAATAGTTGT-3’ |  | This study |
| CassetF |  | 5’-GCTTTCGGTCGCTGTTCTCA-3’ |  | This study |
| CassetR |  | 5’-TGTTAGGCGTCATCCTGTGC-3’ |  | This study |
| ATP1-5  ATP1-6  ATP1-7  ATP1-8  ATP1-F  ATP1-R  ATP1-11  ATP1-12 |  | 5’-GATGACTTGCGTCGTATC-3’  5’-CCAGACAGTCGAGTTAGAC-3’  5’-ACTAAGCAGACAGCTCCTTG-3’  5’-AATAGACGATGACGACGACT-3’  5’-TTTCCCTCCTCCGCACAT-3’  5’- GAGCAATACCATCCCTAA-3’  5’-TTGATGACTTGCGTCGTATC-3’  5’-AGTTGCCTGATGAAGGAGTT-3’ |  | This study  This study  This study  This study  This study  This study  This study  This study |

^a^ Underlined sequences represent the following restriction sites: ApaI for ATP1-1, XhoI for ATP1-2, NotI for ATP1-3, SacII for ATP1-4.

**Table S2｜**Susceptibilities of azole against *C. albicans* by microdilution assay.

| Strains | MIC_80_ (range) of drugs(μg/ml) | | | |
| --- | --- | --- | --- | --- |
|  | FLC | ITR | VRC | KCZ |
| WT | 0.25 | 0.125 | 0.0625 | 0.0625 |
| *atp1*△/△ | 0.50 | 0.125 | 0.0625 | 0.0625 |
| *atp1*△/*ATP1* | 0.25 | 0.125 | 0.0625 | 0.0625 |

**Table S3｜**Doubling times of *C. albicans* in the exponential growth phase at 30℃.

|  | Doubling times (h) |
| --- | --- |
| WT | 2.04 |
| *atp1*△/△ | 2.35 |
| *atp1*△/*ATP1* | 2.01 |


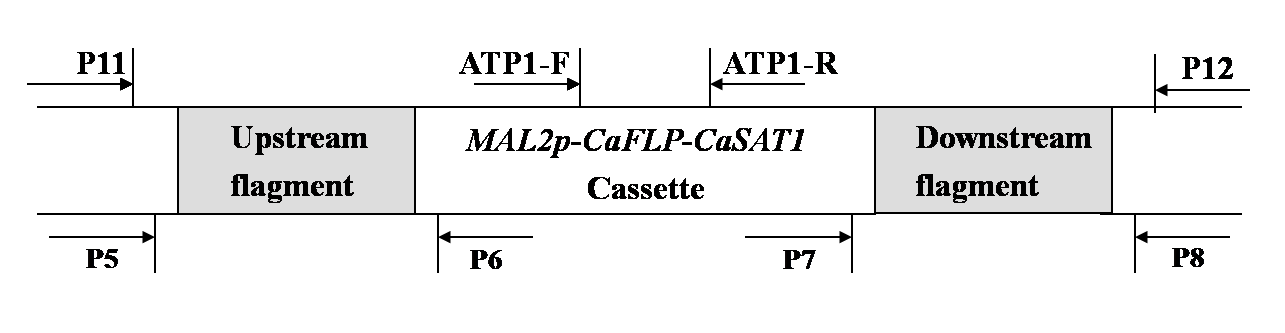


**Figure S1｜Schematic diagram of the primers used to identify the disruption of target gene.**


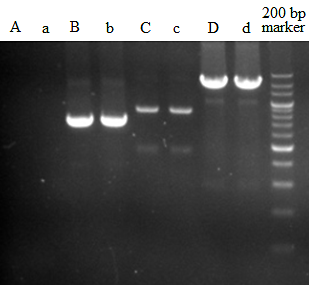


**Figure S2｜Identification of the *ATP1* knockout mutant and reconstituted strain.** Primers used in this work were *ATP1*inside Check and outside Check. Amplified with primer ATP1-F+ATP1-R, A is without stripe for *atp1*Δ/Δ, B and b are stripesat 1380bpfor WT and *atp1*Δ/*ATP1,* respectively. Amplified with primer P11+P12, C is stripe at 1516bpfor *atp1*Δ/Δ, D is stripe at 3659bp for WT. These results suggest the ATP1 null mutant and reconstituted strain are constructed successfully.


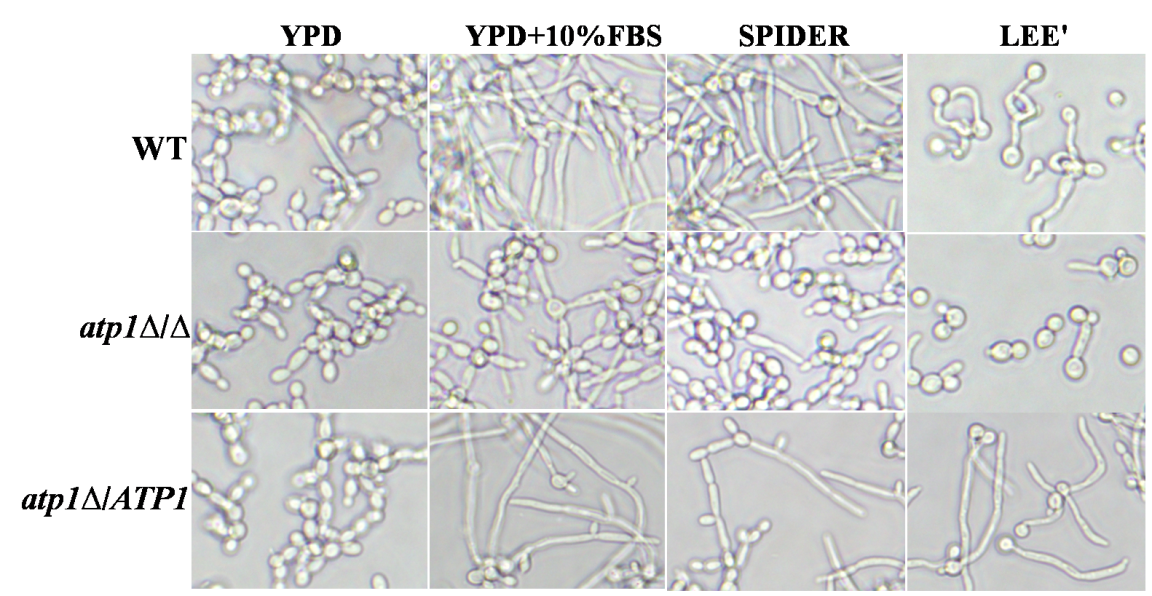


**Figure S3｜Deletion of *ATP1* displays defective filamentation.** Overnight cultures of the strains were resuspended to 1.0 × 10^6^ cells/ml, and added into 12-well microtiter plates with filaments-inducing media. All the plates were cultured at 37℃ for 4 hours before photographed.


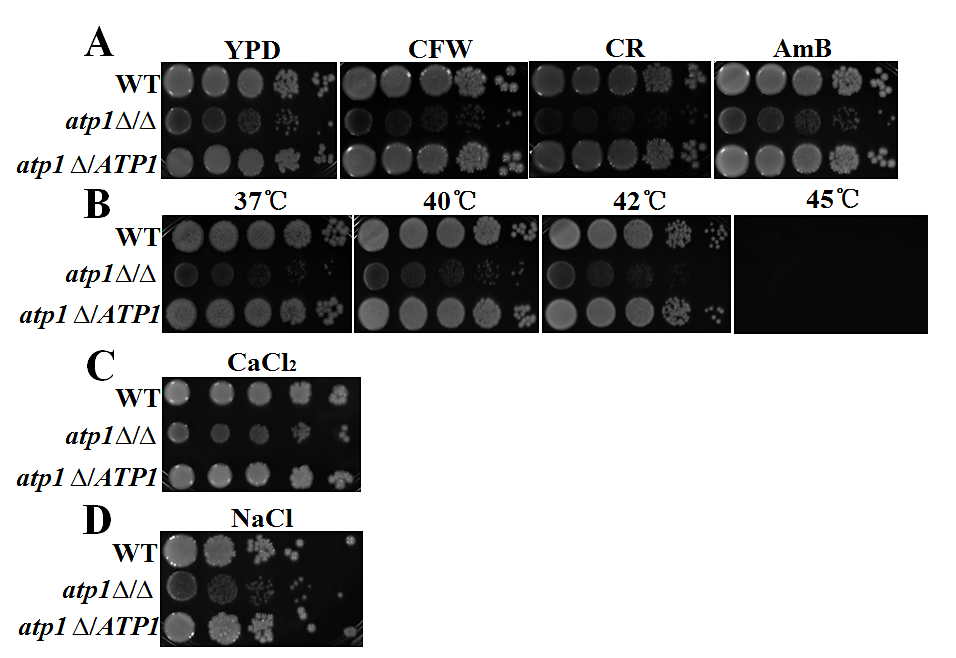


**Figure S4｜**(A) Susceptibility to agents that effect on cell wall. Strains were grown in the presence of 40 µg/ml Calcofluor white (CFW), 25 µg/ml Congo red (CR) and 0.5 µg/ml AmB. (B) Susceptibility to heat of 40℃, 42℃ and 45℃. (C) Susceptibility to calcium stress 125 mM CaCl2. (D) Susceptibility to osmotic stress (1 M NaCl).


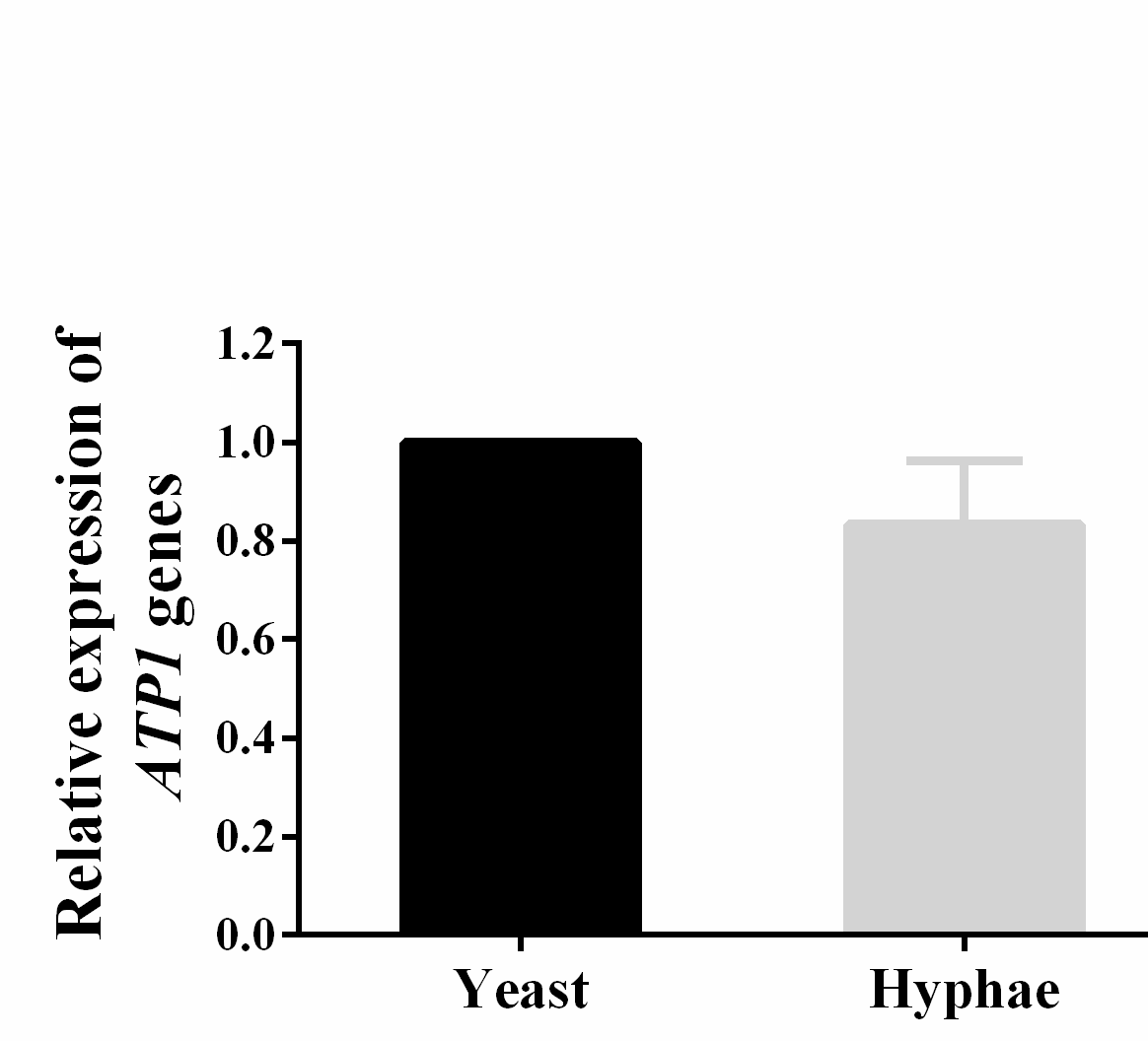


**Figure S5｜The expression of *ATP1* between in yeast form and hyphal form.** WT was incubated in YPD, Spider media at 37°C for 6 hours. Following incubation expression of the indicated genes were determined by qRT-PCR.
